# Upregulated complement receptors correlate with Fc gamma receptor 3A-positive natural killer and natural killer-T cells in neuromyelitis optica spectrum disorder

**DOI:** 10.1186/s12974-022-02661-1

**Published:** 2022-12-12

**Authors:** Shuhei Nishiyama, Amy E. Wright, Itay Lotan, Takahisa Mikami, Friedemann Paul, Masashi Aoki, Michael Levy

**Affiliations:** 1 Department of Neurology, Massachusetts General Hospital, Building 114, 16th St., Room 3150, Charlestown, MA 02129 Boston, USA; 2 Harvard Medical School, Boston, MA USA; 3 Department of Neurology, Tohoku University Graduate School of Medicine, Sendai, Miyagi Japan; 4 Department of Neurology, Tufts University School of Medicine, Boston, MA USA; 5 Experimental and Clinical Research Center, Max Delbrück Center for Molecular Medicine and Charité-Universitätsmedizin Berlin, Corporate Member of Freie Universität Berlin and Humboldt-Universität zu Berlin, Berlin, Germany

## Abstract

**Background and objectives:**

Inhibition of terminal complement in neuromyelitis optica spectrum disorder (NMOSD) using eculizumab helps prevent relapses, but the exact mechanism of action of the drug remains unclear. Similarly, genetic variants in the Fc Gamma receptor 3A (*FCGR3A*), also known as CD16, are correlated with outcomes in NMOSD, but the immune cells expressing those CD16 are unknown. We compared CD16 expression on immune cells modulated by complement activity in natural killer (NK) cells and natural killer-T (NKT) cells in NMOSD to disease and normal-healthy controls.

**Methods:**

Peripheral blood cell (PBMC) samples from 45 patients with NMOSD with aquaporin 4 (AQP4)-IgG, 18 disease controls, and 19 normal controls were analyzed for CD16 expression and complement receptors in vitro.

**Results:**

At baseline, the number of NKT cells was increased in NMOSD (*p* < 0.001), but the proportion that was CD16 positive was lower compared to normal and disease controls (*p* = 0.0012). NK cell count was normal, but the ratio that was CD16 positive was also significantly lower (*p* < 0.001). In both NK cells and NKT cells from NMOSD, C5 complement receptor expression was much higher than normal and disease controls (*p* < 0.001 for both). We also evaluated activation markers CD69 and CD83, which were also significantly higher in NK and NKT cells from NMOSD patients. *FCGR3A p158* V/V genotype group in NMOSD patients showed decreased NK cell proportion with activation, and fewer CD16-expressing NKT cells than the F/F genotype group.

**Discussion:**

Our results support an immunopathogenesis model in which complement pathway activation in NK/NKT cells upregulates CD16 expression that binds to antibody/antigen complexes. In the context of NMOSD, these complement-sensitive cells may be responsible for the escalating autoimmune activity.

**Supplementary Information:**

The online version contains supplementary material available at 10.1186/s12974-022-02661-1.

## Introduction

The immunopathogenesis of neuromyelitis optica spectrum disorder (NMOSD) has been shown to involve many components of the adaptive immune system including T cells [1, 2], B cells [3], monocytes [4], as well as the innate immune system, including complement [5], granulocytes [6, 7], plasmablasts [8], and antibodies [9]. In a simplistic model, upstream communication among peripheral T cells, B cells and monocytes leads to a coordinated decision to attack the central nervous system. A breach of the blood brain barrier by aquaporin-4-reactive T cells likely initiates the attack, then the T cells recruit granulocytes like neutrophils and eosinophils and ultimately aquaporin-4 antibodies that can fix complement and destroy astrocytes and nearby myelin [10].

Human trials of the C5 terminal complement inhibitor, eculizumab, proved very effective in preventing relapses [11]. Immunopathogenesis models would have predicted that blocking the final step of the complement cascade would have prevented membrane attack complex (MAC) complex formation and downstream astrocyte injury within the central nervous system (CNS). If the C5 inhibitor only inhibits complement-dependent astrocytic necrosis via AQP4-IgG, it would not explain the relapse rate reduction. Thus, the mechanism of action in C5 inhibitors may manipulate upstream peripheral immunological activity beginning with C5a to prevent the initiation of an attack.

In another human trial in NMOSD testing, the efficacy of a CD19 monoclonal B cell depletion strategy to prevent relapses, beyond the expectation that B cell depletion was effective in preventing relapses [12]. An interesting discovery was reported that did not gain widespread attention at the time: a genetic variant in the Fc gamma receptor 3A gene (*FCGR3A*, position 158), encoding CD16A, significantly predicted outcomes in the placebo arm [13]. The genetic variation at position 158 predicts how the receptor will bind the Fc portion of antibodies, with the phenylalanine (F) F/F homozygous genotype binding the weakest, the valine (V) V/V binding the strongest and the V/F genotype in between. In this trial of NMOSD participants not on any monoclonal therapies, the F/F genotype had the best clinical outcomes and the V/V genotype had the worst, with V/F in between. This was the first study that showed a significant genetic impact on outcomes in NMOSD. Known to be expressed by NK cells and monocytes, we do not understand how *FCGR3A* influences the immunopathogenesis of NMOSD.

To begin to investigate the upstream immunological processes that lead to NMOSD attacks, we focused on the convergence of CD16 expression and complement activity at the level of NK and NKT cells. As effectors of innate immunity, NK cells can quickly react to infections and cancers without the requirement of self-major histocompatibility complex (MHC) class I signals or antibodies. NK cells can also behave as adaptive immune cells with antigen-specificity and immunological memory where they may be involved in autoimmunity [14]. NKT cells are much less numerous than NK cells, but they serve many of the same functions with the additional capability of signaling with CD1d molecules that can present self-antigens and contribute to autoimmunity [15]. Although there have been some previous reports studying the prevalence of NK and NKT cells in NMOSD so far [16–18], no studies have been reported that have performed a comprehensive analysis including the *FCGR3A* polymorphism. Because both NK and NKT cells express CD16A encoded by *FCGR3A* and react to complement activity, we sought to characterize the levels and patterns of expression in NMOSD compared to healthy and disease controls.

## Materials and methods

### Patients and Peripheral blood mononuclear cells (PBMCs)

PBMCs from serum aquaporin-4 (AQP4)-IgG-positive NMOSD patients measured by cell-based assay (CBA) were donated from The Guthy-Jackson Foundation and Prof. Friedemann Paul. All NMOSD samples were from patients in remission with a gap of at least 4 weeks between sample collection and the date of last relapse. The PBMCs were isolated by Ficoll's method. Disease controls (DC) and normal controls (NC) were obtained from the Guthy-Jackson Charitable Foundation and healthy volunteers under informed consent. Forty-five random NMOSD with AQP4-IgG patients, 18 disease controls, and 19 normal controls were included in this cohort. Disease controls include eight with myelin-oligodendrocyte glycoprotein (MOG)-IgG associated disease (MOGAD) [19], four with a diagnosis multiple sclerosis (MS) who were seronegative for both AQP4 and MOG-IgG, and one of each following diagnoses: Crohn's disease, neuropsychiatric systemic lupus erythematosus, rheumatoid arthritis, transverse myelitis, ankylosing spondylitis, and AQP4-IgG-seronegative NMOSD. The demographics of study participants are shown in Table 1.

**Table 1:** Profiles of each group in the cohort

	NMOSD	Disease controls	Normal controls
	(*n *= 45)	(*n *= 18)	(*n *= 19)
Age	45.79 ± 14.98	44.7 ± 19.33	43.67 ± 15.61
Sex (M:F)	5:40	3:15	6:13
AQP4-IgG serostatus	100% (45/45)	0% (0/18)	0% (0/19)

### Flow cytometry and data analysis

BD Fortessa X-20 (BD Bioscience) was used for flow cytometry analysis. After doublet cells were excluded, lymphocytes and monocytes fractions were isolated by plotting forward- and side-scatter heights. The following antibodies were used for the assay: CD3-AF700 (BioLegend), CD11b-BV421 (BD Bioscience), CD14-BV785 (BioLegend), CD14-PerCP-Cy5.5 (BioLegend), CD16-BUV395 (BD Bioscience), CD35-VioBlue (Miltenyi Biotec), CD35-PE (BioLegend), CD45-BUV737 (BD Bioscience), CD45-BV711 (BioLegend), CD56-BV510 (BioLegend), CD69-BV650 (BioLegend), CD83-BV605 (BioLegend), CD88-APC (BioLegend), CD88-BV786 (BD Bioscience), CX3CR1-PE (BioLegend), and TCR Vα24-Jα18-APC-Vio770 (Miltenyi Biotec). LIVE/DEAD™ Fixable Blue Dead Cell Stain Kit (Thermo Fisher Scientific) was used to exclude dead cells from peripheral blood mononuclear cells (PBMCs). After Fc receptor blocking using FcR Blocking Reagent (Immunostep) according to the manufacturer’s instruction, PBMCs were stained with the surface markers for 30 min at 4 °C. They were then fixed with 4% paraformaldehyde, permeabilized with 0.1% Tween-20. Cells were stained with intracellular markers for 30 min at 4 °C in PBS with 0.5% fetal bovine serum (FBS) and 2 mM of ethylenediaminetetraacetic acid (EDTA). NK, NKT and monocytes are defined as follows: NK (CD45 + CD14-CD3-CD56 +), NKT (CD45 + CD14-CD3 + CD56 +), and monocytes (CD45 + CD11b + (CD14 + and/or CD16 +)). The gating strategy in the study is shown in Additional file 1: Fig. S1.

### Polymerase chain reaction (PCR)

The DNAs were extracted from PBMC samples with QIAamp DNA Micro (Qiagen, USA). GoTaq Green Master Mix (Promega, USA) and primers were mixed as per the manufacturer's instructions. The primer sequences used in the reaction are as follows: *FCGR3A* common forward primer (TCC AAA AGC CAC ACT CAA AGT C), *FCGR3A p158* V reverse primer (AGA CAC ATT TTT ACT CCC ATC), and *FCGR3A p158* F reverse primer (AGA CAC ATT TTT ACT CCC ATA). After incubation of 95 °C for 5 min, 35 cycles of 95 °C for 20 s, 56 °C for 20 s, and 72 °C for 30 s were performed.

### Statistical analysis

Data were analyzed with FlowJo v10.7.1 (Becton Dickinson & Company) and GraphPad Prism 8.4.3 (GraphPad Software, LLC). The groups were compared using the Kruskal–Wallis test, and Spearman’s rank correlation was used for the analysis of correlations between parameters. Due to the exploratory nature of the study, no adjustment for multiple comparisons was made. A statistical significance was defined as *p* < 0.05.

## Results

**Fig. 1:**
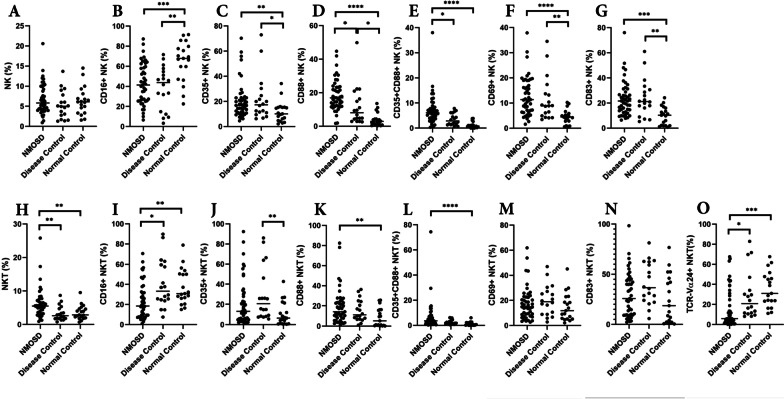
Flow cytometry analysis of NK and NKT cells. Although there is no change in the abundance of NK cells themselves (**A**), CD16-positive NK cells are significantly decreased in NMOSD (**B**). Complement receptors CD35 (**C**) and CD88 (**D**) were increased in NMOSD, and CD35 + CD88 + co-positive cells were markedly increased (**E**). Activation markers CD69 (**F**) and CD83 (**G**) were also increased in NK cells. Analysis of NKT cells showed they were significantly increased in NMOSD (**H**), while CD16-positive cells (**I**) and TCR Vα24-positive cells (**O**) were significantly decreased. CD88 (**K**) and CD35 + CD88 + double-positive (**L**) showed significant increases compared to NC, while CD35 (**J**) and activation markers (**M**, **N**) were unchanged. NMOSD (*n* = 45), disease controls (*n* = 18), and healthy controls (*n* = 19). *p < 0.05; **p < 0.01; ***p < 0.001; ****p < 0.0001

The percentage of NKT cells as a proportion of all white blood cells was increased in the NMOSD group compared to the other groups (NMOSD 6.219 ± 4.350%, DC 3.322 ± 2.192%, NC 3.576 ± 2.124%; *p* = 0.0006) (Fig. 1H). Among them, the percentage of NKT cells expressing CD16 in NMOSD was lower than in the other groups (NMOSD 23.33 ± 17.98%, DC 40.64 ± 24.17%, NC 37.65 ± 17.55%; *p* = 0.0012) (Fig. 1I). In contrast, the percentages of NK cells in the NMOSD, DC, and NC were 6.807 ± 3.841%, 5.413 ± 3.463%, and 6.377 ± 3.461%, respectively, without significant differences (Fig. 1A). Similar to NKT cells, the proportion of NK cells expressing CD16 was lower in the NMOSD group compared to NC (NMOSD 42.71 ± 20.40%, NC 64.87 ± 19.98%; *p* = 0.0004) and was also significantly lower in the disease controls (DC 38.43 ± 21.44%) (Fig. 1B).

The classical NKT, also known as invariant NKT (iNKT), play a significant role in all NKT cells. iNKT has invariant TCR Vα24-Jα18, and is CD1d-restricted. We analyzed the proportion of iNKT among NKT using the TCR Vα24-Jα18 antibody. Results showed the percentage of TCR Vα24-Jα18-positive NKT was lower in the NMOSD than in the DC and NC groups (NMOSD 16.50 ± 19.60%, DC 29.12 ± 23.64%, NC 35.28 ± 17.36%; *p* = 0.0001) (Fig. 1O). We also analyzed the surface marker expressions in TCR Vα24-Jα18-positive NKT cells. The only significant result is obtained from CD83-positive subsets, which showed significantly higher in the NMOSD group than the Normal Control group (Additional file 2: Fig. S2).

**Fig. 2:**
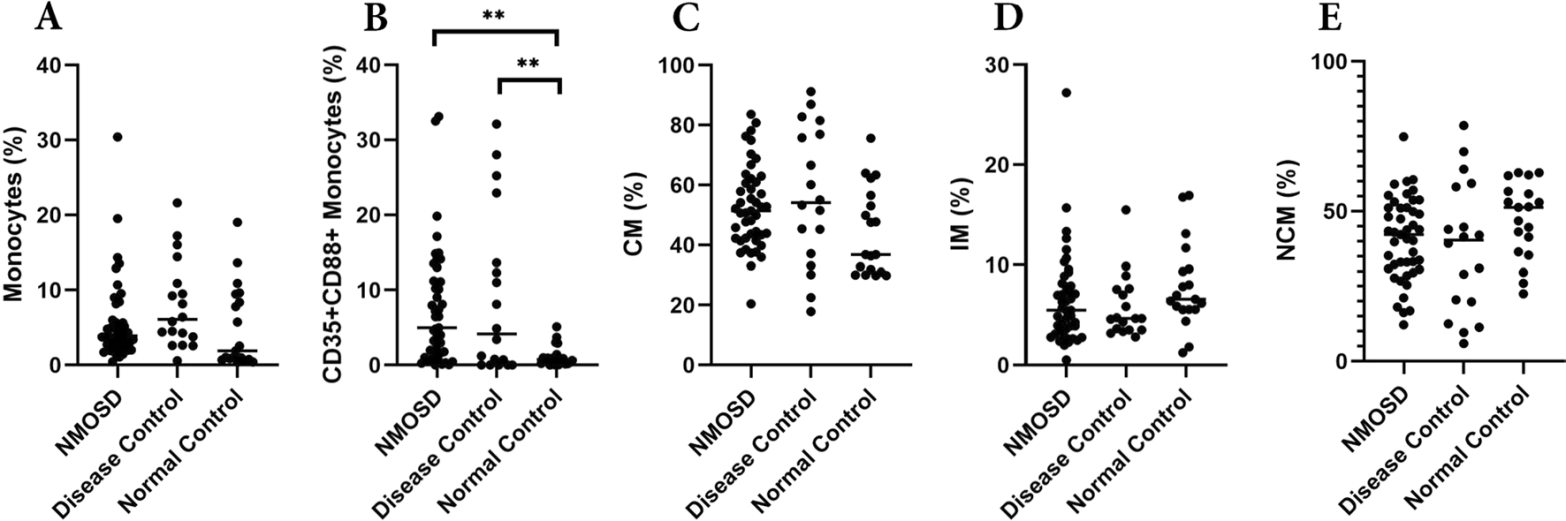
Flow cytometry analysis of monocytes. There are no significant differences between the percentage of monocytes (**A**), classical monocytes (**C**), intermediate monocytes (**D**), and non-classical monocytes (**E**). Complement receptors in monocytes are significantly upregulated in NMOSD and DC (**B**). NMOSD (*n* = 45), disease controls (*n* = 18), and Healthy controls (*n* = 19). **p < 0.01

We simultaneously analyzed monocyte populations in each cohort. The percentages of monocytes in NMOSD, DC, and NC were 9.383 ± 7.833%, 11.26 ± 6.592%, and 8.042 ± 5.329%, respectively, with no statistically significant differences between the groups (*p* = 0.5004) (Fig. 2A). The proportions of classical monocytes (CM), intermediate monocytes (IM), and non-classical monocytes (NCM) among monocytes was not different either (Fig. 2C–E). Unlike NK and NKT cells in NMOSD, CD16 expression on monocytes was not different in NMOSD compared to controls (Fig. 2E, F).

We then focused on the surface expression of two complement receptors in NK and NKT cells: CD35 (CR1), the receptor for C3b and C4b, and CD88 (C5aR), the receptor for C5a. There was a significant increase in CD35-positive NK cells in NMOSD and DC compared to NC (NMOSD 20.89 ± 14.68%, DC 22.02 ± 18.09%, NC 11.63 ± 8.240%; P = 0.0083). In NKT cells, CD35 expression was higher in both NMOSD and disease controls, compared to healthy controls (NMOSD 22.08 ± 22.78%, DC 30.03 ± 26.82%, NC 10.74 ± 11.84%; P = 0.0086) (Fig. 1C, J). The C5a receptor, CD88, was elevated in NMOSD in both NK and NKT cells (CD88 + NK: NMOSD 7.184 ± 6.017%, DC 3.487 ± 2.365%, NC 1.193 ± 1.233%; P < 0.0001, CD88 + NKT: NMOSD 6.814 ± 11.68%, DC 2.765 ± 1.812%, NC 1.292 ± 1.537%; *p* = 0.0001) (Fig. 1D, K). Of note, the number of CD35 + CD88 + double-positive cells was significantly elevated in both NK and NKT cells in the NMOSD group (CD35 + CD88 + NK: NMOSD 7.184 ± 6.017%, DC 3.487 ± 2.365%, NC 1.193 ± 1.233%; P < 0.0001, CD35 + CD88 + NKT: NMOSD 6.814 ± 11.68%, DC 2.765 ± 1.812%, NC 1.292 ± 1.537%; *p* = 0.0001) (Fig. 1E, L). Monocytes from NMOSD patients also expressed elevated levels of complement receptors, but they were also elevated on monocytes in other diseases (Fig. 2B).

We analyzed the expression of CD69 as a marker of early activation of NK and NKT cells. CD69 expression was significantly increased in NMOSD compared with NC, while an increase in CD69 + NK was also observed in DC compared with NC (NMOSD 12.96 ± 7.721%, DC 11.55 ± 8.765%, NC 4.543 ± 2.873%; *p* < 0.0001) (Fig. 1F). In contrast, there was no significant difference in CD69 expression on NKT cells among NMOSD versus healthy or disease controls (Fig. 1M). CD83 is another marker found on activated T and B cells, circulating dendritic cells, Langerhans cells, macrophages, monocytes, neutrophils, and NK cells [20]. There was a significant increase in CD83-positive cells in NMOSD and DC in NK cells (NMOSD 23.49 ± 13.83%, DC 24.14 ± 15.06%, NC 9.744 ± 7.248%; *p* = 0.0001) (Fig. 1G). The percentage of CD83 + NKT cells in each cohort was similar to CD69 (NMOSD 29.71 ± 22.21%, DC 41.26 ± 21.25%, NC 25.44 ± 24.05%; *p* = 0.0553) (Fig. 1N). A summary of the analyzed flow cytometry data is shown in Table 2.

**Table 2:** Percentages of positivity for cell surface markers of NK cells, NKT cells, and monocytes in each group

(%)	NMOSD	Disease controls	Normal controls	*p*
	(*n *= 45)	(*n *= 18)	(*n *= 19)	
NK Cells	6.807 ± 3.841	5.413 ± 3.463	6.377 ± 3.461	0.3827ns
CD16+	42.71 ± 20.40	38.43 ± 21.44	64.87 ± 19.98	0.0004***
CD35+	20.89 ± 14.68	22.02 ± 18.09	11.63 ± 8.240	0.0083**
CD88+	18.59 ± 9.319	11.86 ± 11.73	3.966 ± 3.925	< 0.0001****
CD35+CD88+	7.184 ± 6.017	3.487 ± 2.365	1.193 ± 1.233	< 0.0001****
CD69+	12.96 ± 7.721	11.55 ± 8.765	4.543 ± 2.873	< 0.0001****
CD83+	23.49 ± 13.83	24.14 ± 15.06	9.744 ± 7.248	0.0001***
NKT Cells	6.219 ± 4.350	3.322 ± 2.192	3.576 ± 2.124	0.0006***
CD16+	23.33 ± 17.98	40.64 ± 24.17	37.65 ± 17.55	0.0012**
CD35+	22.08 ± 22.78	30.03 ± 26.82	10.74 ± 11.84	0.0086**
CD88+	19.35 ± 17.36	15.33 ± 10.76	8.759 ± 9.246	0.0112*
CD35+CD88+	6.814 ± 11.68	2.765 ± 1.812	1.292 ± 1.537	0.0001***
CD69+	17.77 ± 13.11	20.17 ± 12.21	14.81 ± 11.27	0.3159ns
CD83+	29.71 ± 22.21	41.26 ± 21.25	25.44 ± 24.05	0.0553ns
TCR Vα24+	16.50 ± 19.60	29.12 ± 23.64	35.28 ± 17.36	0.0001***
Monocytes	5.780 ± 5.473	8.038 ± 5.905	5.048 ± 5.499	0.2295ns
CD35+CD88+	7.478 ± 7.732	9.122 ± 10.95	1.146 ± 1.420	0.0030**
CM	52.72 ± 13.85	56.29 ± 22.89	44.49 ± 14.53	0.0775ns
IM	6.507 ± 4.608	5.850 ± 3.181	7.863 ± 4.276	0.3345ns
NCM	40.77 ± 13.80	37.86 ± 22.03	47.20 ± 12.63	0.1756ns

*n.s.* not significant, *p < 0.05; **p < 0.01; ***p < 0.001; ****p < 0.0001

**Fig. 3:**
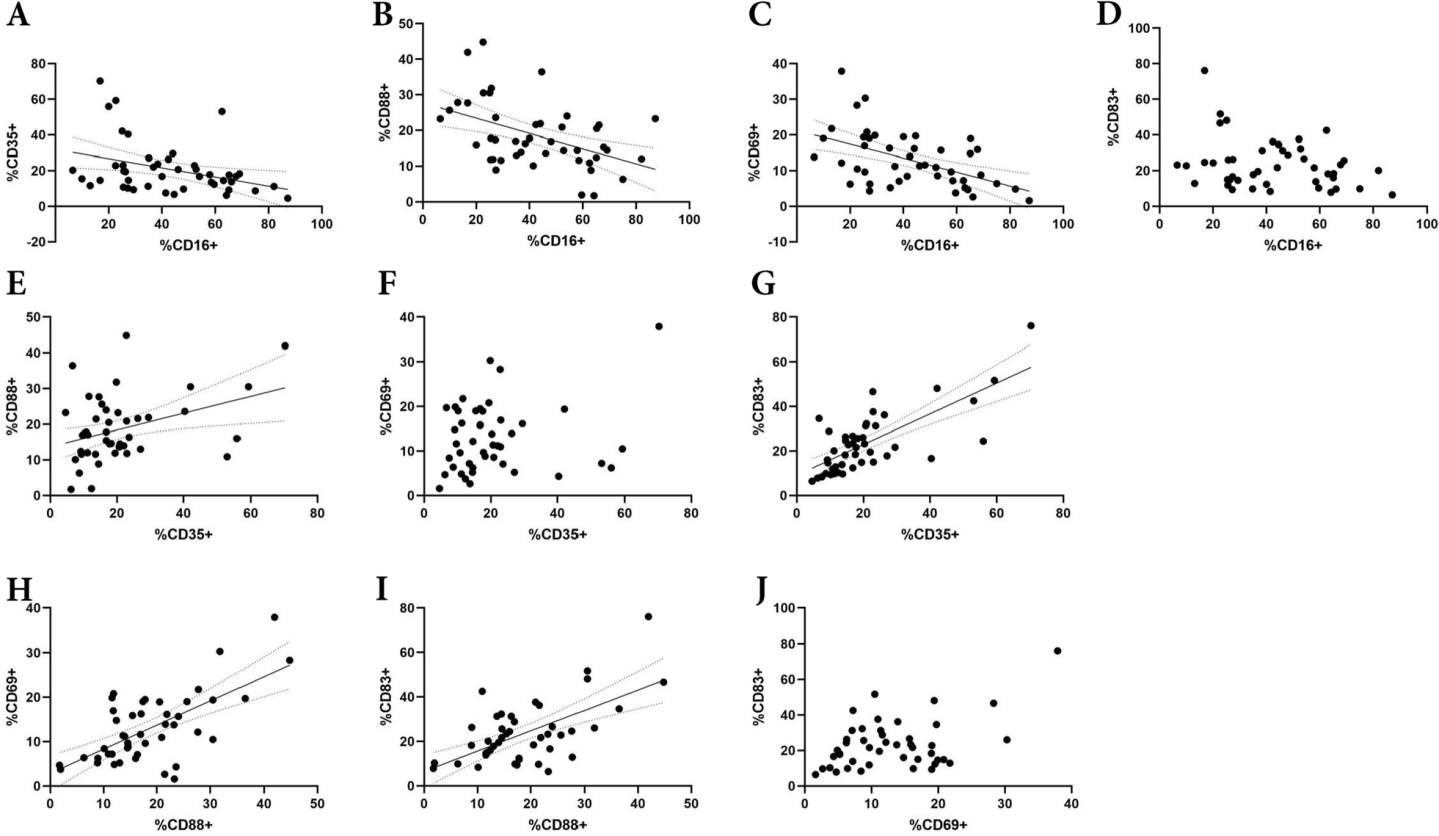
Spearman correlation analysis in NK cells of NMOSD group (*n* = 45). Dot plots show the results of correlation analysis between parameters each other: CD16 vs CD35 (**A**), CD16 vs CD88 (**B**), CD16 vs CD69 (**C**), CD16 vs CD83 (**D**), CD35 vs CD88 (**E**), CD35 vs CD69 (**F**), CD35 vs CD83 (**G**), CD88 vs CD69 (**H**), CD88 vs CD83 (**I**), and CD69 vs CD83 (**J**). There is a significant positive correlation between CD35 and CD83 (**G**). There was a negative correlation between CD16 and CD35 (**A**), CD88 (**B**), and CD69 (**C**)

**Fig. 4:**
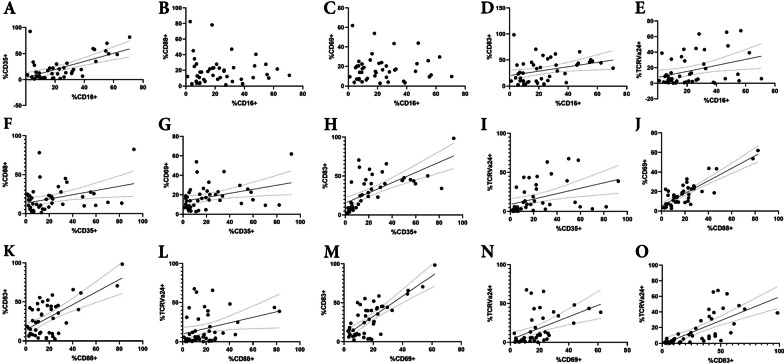
Spearman correlation analysis in NKT cells of NMOSD group (*n* = 45). Dot plots also show the results of correlation analysis between parameters each other: CD16 vs CD35 (**A**), CD16 vs CD88 (**B**), CD16 vs CD69 (**C**), CD16 vs CD83 (**D**), CD16 vs TCR Vα24 (**E**), CD35 vs CD88 (**F**), CD35 vs CD69 (**G**), CD35 vs CD83 (**H**), CD35 vs TCR Vα24 (**I**), CD88 vs CD69 (**J**), CD88 vs CD83 (**K**), CD88 vs TCR Vα24 (**L**), CD69 vs CD83 (**M**), CD69 vs TCR Vα24 (**N**), and CD83 vs TCR Vα24 (**O**). There is a significant positive correlation between CD35 and CD83 (**H**). CD16 showed negative correlation between CD35 (**A**), CD88 (**B**), and CD69 (**C**). A positive correlation was observed between TCR Vα24 positivity and CD35 (**I**), CD69 (**N**) and CD83 (**O**). Positive correlations were also observed between activation markers and complement receptors, namely between CD35 and CD83 (**H**), and CD88 and CD69 (**J**)

We performed Spearman correlation analysis for the NMOSD group to examine potential correlations between the parameters (Additional file 3: Table S1). Moderate positive correlations were found between CD35 and CD83 (*p* < 0.0001, *r* = 0.6482) (Fig. 3G), and CD88 and CD69 (*p* = 0.0004, *r* = 0.5040) in NK cells (Fig. 3H). Weak-negative correlations were also seen between CD16 and CD35 (Fig. 3A), CD16 and CD88 (Fig. 3B), and CD16 and CD69 (Fig. 3C). No correlation was observed between CD16 and CD83 (Fig. 3D), CD35 and CD69 (Fig. 3F), and CD69 and CD83 (Fig. 3J). The same analysis was performed for NKT cells, with significant strong positive correlations between CD88 and CD69 (*p* < 0.0001, *r* = 0.7103) (Fig. 4J), CD35 and CD83 (*p* < 0.0001, *r* = 0.7900) (Fig. 4H), and CD83 and TCR Vα24-Jα18 (*p* < 0.0001, *r* = 0.7951) (Fig. 4O). There were also moderate positive correlations between the following combinations: CD35 and TCR Vα24-Jα18 (Fig. 4I), CD69 and CD83 (Fig. 4M), and CD69 and TCR Vα24-Jα18 (Fig. 4N). Weak-positive correlations were detected between CD16 and CD35 (Fig. 4A), and CD69 and CD35 (Fig. 4G), but no correlation was observed between CD16 and CD88 (Fig. 4B), and CD16 and CD69 (Fig. 4C).

**Fig. 5:**
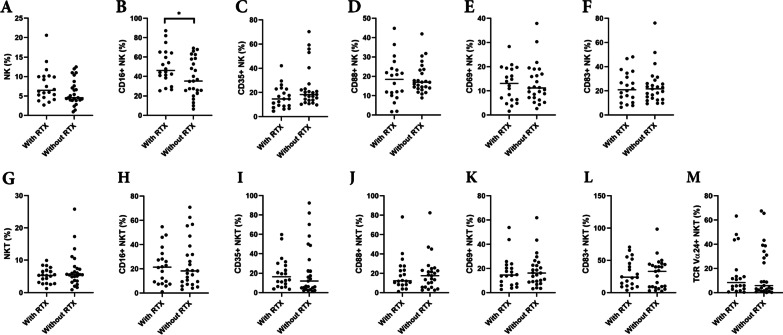
Analysis of parameters with and without rituximab in NMOSD (*n* = 45). The percentage of NK cells (**A**–**F**) and NKT cells (**G**–**M**) is shown among NMOSD patients with (left column) or without (right column) rituximab intervention. Only CD16 + NK cells significantly increased NMOSD with rituximab (**B**). *p < 0.05

Among the NMOSD group who were using the B cell depletion rituximab as preventive therapy (*n* = 20), the only impact was an increase in CD16 expression on NK cells in rituximab-treated patients compared to the patients without rituximab (*n* = 25) (Fig. 5B). No significant differences were found between the NMOSD patients with and without rituximab in the other parameters (Additional file 4: Table S2). Finally, we analyzed the correlation between the number of days from the date of sample collection to relapse date and each parameter, but found no significant correlations (data not shown).

**Fig. 6:**
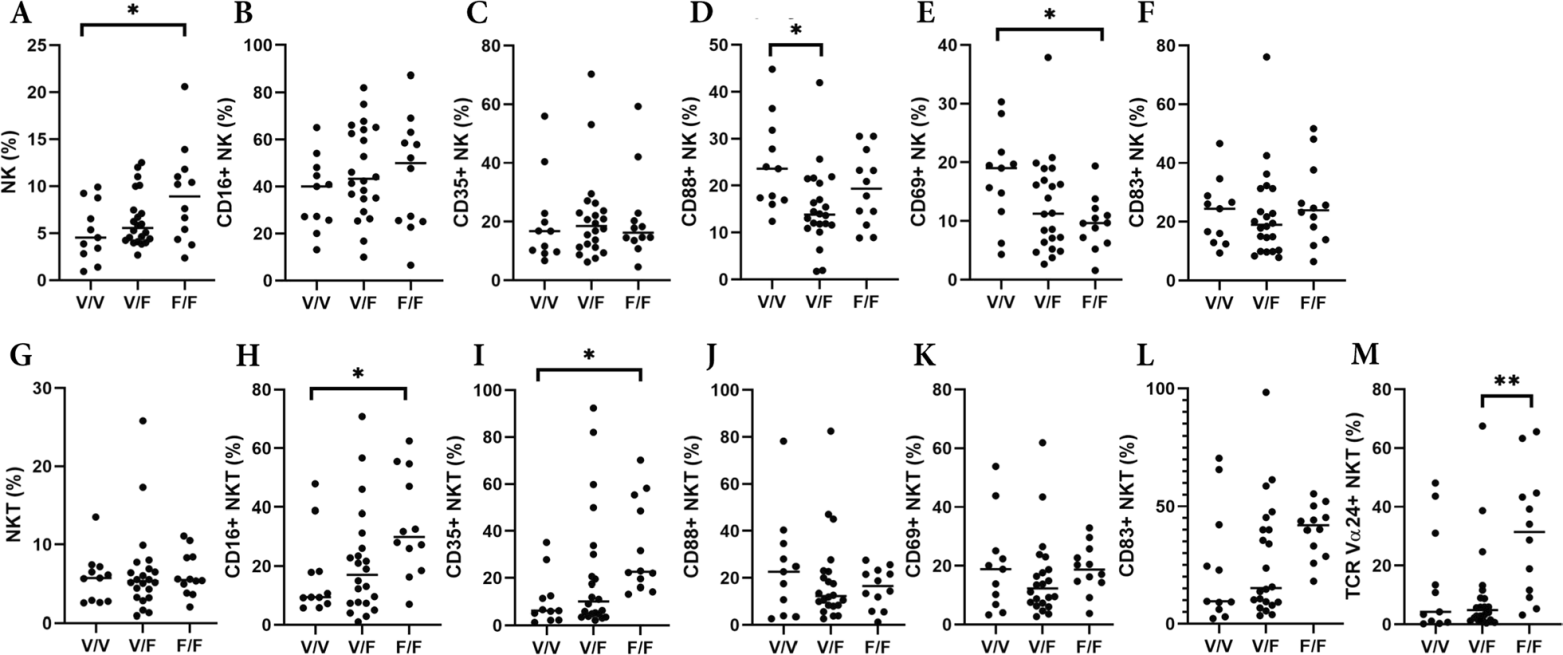
Analysis of parameters among *FcGR3A p158* polymorphisms in NMOSD. The percentage of NK cells (**A**–**F**) and NKT cells (**G**–**M**) is shown among NMOSD patients with V/V, V/F, and F/F genotypes. The ratio of NK cells in total PBMCs was significantly decreased in the V/V genotype group compared to the F/F group (**A**). There was no difference in the expression of CD16 (**B**), CD35 (**C**), and CD83 (**F**) in these cells, but CD69-positive NK cells were significantly increased (**E**), and CD88-positive NK cells were significantly increased in the V/V group compared with the V/F group (**D**). Meanwhile, there was no difference in the ratio of NKT cells (**G**), but CD16-positive NK cells were decreased in the V/V group (**H**). Furthermore, TCR Vα24-positive NKT cells were significantly increased in the F/F group compared to the V/F group (**M**), and there was also a tendency for an increase in the F/F group compared to the V/V group (*p* = 0.0861). The expression of CD35 in NKT cells was also significantly decreased in V/V group than F/F group (**I**). There was no difference in complement receptors or activation markers on the surface of NKT cells between the groups (**J**–**L**). *p < 0.05; **p < 0.01

We further analyzed the effect of *FCGR3A p158* polymorphism on NK cell ratio and its surface markers in NMOSD patients. 45 patients had V/V, V/F, and F/F genotypes in 11, 22, and 12 patients, respectively. The ratio of NK cells among all PBMCs in the V/V genotype group was significantly lower than that in the F/F group (*p* = 0.0400, Fig. 6A). However, their ratio in the V/F group was not significantly different from that in the other groups (V/V vs. V/F: *p* = 0.6119; V/F vs F/F: *p* = 0.1346) and was intermediate between the V/V and F/F groups. The number of CD69-positive NK cells was significantly increased in the F/F group compared with the V/V group (*p* = 0.0388, Fig. 6E). The expression of CD88 on NK cells was significantly upregulated in the V/V group compared with the V/F group (*p* = 0.0175, Fig. 6D). The same analysis was performed for NKT cells. There was no significant difference in the expression of CD16 (Fig. 6B), CD35 (Fig. 6C), and CD83 (Fig. 6F) in NK cells. Although there was no difference in the ratio of NKT cells (Fig. 6G), the ratios of CD16-positive NK cells (Fig. 6H) and CD35-positive NK cells (Fig. 6I) in the V/V group were significantly lower than those in the F/F group, respectively. In addition, TCR Vα24-positive NKT cells were significantly increased in the F/F group compared to the V/F group (*p* = 0.0071, Fig. 6M), and there was an increasing trend in the F/F group compared to the V/V group (*p* = 0.0861).

## Discussion

In this study, we have focused on the convergence of complement reactivity and CD16 expression in immune cells that may be involved in the pathogenesis of NMOSD. If terminal complement inhibition only acted to protect against downstream MAC formation at the astrocyte endfoot, it would not have prevented relapses from initiating. Therefore, C5 complement inhibition with eculizumab in NMOSD trials must be acting upstream of the complement MAC complex as this drug proved remarkably effective in preventing relapses from occurring at all [11]. Similarly, the impact of a genetic variation in *FCGR3A* in NMOSD outcomes strongly implicates this receptor in the immunopathogenesis of the disease. Among upstream immune targets that are (1) sensitive to peripheral complement activity, (2) can express CD16 and (3) are antigen-specific, monocytes and NK/NKT cells are prime candidates.

We found that the expression of complement receptors, CD16, and immune activation markers in NK and NKT cells are significantly changed in NMOSD patients compared to the control groups. This difference appears to be specific to NK and NKT cells as there is no difference in these markers on monocytes between NMOSD and disease controls. In line with our data showing a decrease of NK populations expressing CD16 and an increase in NKT cells in NMOSD, two previous reports showed that NK cells were decreased in NMOSD [17, 18]. An increase in NKT, as well as a decrease in CD16-expressing NK cells, was previously reported in NMOSD as compared to healthy controls [16]. One possible explanation for this finding is that activated CD16 + NK cells are more likely to migrate out from the circulation. A similar decrease in peripheral circulating NK cells has been reported in primary Sjogren’s syndrome [21]. Interestingly, NKT expressing CD16 were also significantly decreased in our data, suggesting not only NK but also NKT may migrate to peripheral inflammatory tissues. Another possibility for the decrease in NK/NKT cells is that all NMOSD samples used in this study were in remission, and most patients were already on therapeutic intervention; however, this is unlikely as disease controls were also in remission.

Invariant NKT (iNKT) cells all express the same T cell receptor Vα24-Jα18 paired with Vβ11, which binds glycolipid presented by CD1d. In our NMOSD cohort, the invariant NKT cell population was lower compared to healthy and disease controls. The function of iNKT in autoimmune diseases is controversial. There are reports that iNKT can be detrimental when they release interleukin-17 (IL-17) upon stimulation by CD1d or via secreted interleukin-23 (IL-23) from antigen-presenting cells [22]. On the other hand, some reports suggest that iNKT acts in a protective manner on the Th17 lineage by suppressing IL-23 released from monocyte-derived dendritic cells (mDCs) and IL-17 production from memory CD4 + helper T cells [23, 24].

Although small in number, NKT cells play a significant role in autoimmunity through various mechanisms including dendritic cell maturation by antigen presentation, as adjuvants, and through long-term immune memory effects [15]. Also, there are some reports that NK cells act as antigen-presenting cells. HLA-DR expression in NKG2C + adaptive NK cells was upregulated when the cells were incubated with human cytomegalovirus and specific antibodies. CD16 expression on them is simultaneously decreased, whereas CD80/86 molecules have no changes. Notably, chloroquine decreased T-cell response if NK cells were pulsed with HCMV-antibody complex but did not affect NK cells pulsed with HCMV peptide. Antigen presentation by NK cells activated a polyfunctional CD4 + T cell response characterized by degranulation (CD107a) and the secretion of Th1 cytokines (interferon (IFN)-γ and TNF-α) [25]. In the mouse model, NK1.1( +)CD11c( +)CD122( +)MHC class II( +) cells share characteristics with the NK cell lineage and with IFN-producing killer dendritic cells. These cells depend on IL-15 and express E4BP4, cytotoxic and produce type I and type II IFN upon activation and efficient Antigen-presenting cells (APCs) through MHC class II expression and cross-presentation to CD8s [26]. Interestingly, an increased NKT ratio has also been reported in Sjogren’s syndrome [27]. Sjogren's syndrome is one of common coexisting autoimmune diseases of NMOSD [28], indicating these autoimmune diseases may have similar pathogenesis of innate immunity via NKT cells.

Neutrophils are another immune cell that constantly express high levels of C5a the receptor, CD88. C5a-preactivated neutrophils cause glutamate increase of extracellular space of astrocytes, which may be partially responsible for downstream astrocyte damage in acute relapses of NMOSD [29]. Our data suggest that peripheral NK and NKT cells expressing CD88 play another role in the upstream pathogenesis of NMOSD. NK and NKT cells do not usually express CD88, even though CD88-mRNA is present in the nucleus. Nevertheless, they can upregulate CD88 in systemic inflammatory conditions such as *E. coli*-induced sepsis [6].

CD69 is one of the early activation markers of hematopoietic cells, including NK and NKT cells [30]. Stimulation with IL-2, IL-12, TNF-α, and pneumococcal C-polysaccharide increases CD69 expression in NK and NKT cells [30, 31]. Another activation marker, CD83, is known to bind to CD83L on APCs, stabilize MHC class II and CD86, and expand antigen-specific cytotoxic T lymphocytes (CTLs) in vitro if co-expressing with CD80 [32]. Our data indicate a significant increase in the number of activated NKs in NMOSD. Positive correlations were observed between CD35 and CD83, and CD69 and CD88 in both NK and NKT cells, which suggests that complement activity and NK/NKT activation occur at the same time. NK/NKT cell activation can be caused by various stimuli and is not necessarily disease-specific. In other words, any inflammatory activity has the potential to activate NK/NKT cells, and thereby trigger an NMOSD relapse.

CD16-expression on NK cells was significantly decreased in the NMOSD group compared to DC and NC, but not in the rituximab intervention group. The results of the *FCGR3A p158* polymorphism in this study also revealed that they altered the ratio of both NK/NKT cells and the expressions of many cell surface markers. The degree of B cell depletion due to rituximab depends on the *FCGR3A p158* polymorphism and NMOSD patients with the F allele have an increased risk of relapse due to insufficient memory B cell depletion. The suggested reason for this is that the *FCGR3A p158* F/F genotype reduces the affinity of autoantibodies for NK cells and have the slower binding rate, resulting in reduced Antibody-dependent cellular cytotoxicity (ADCC) efficacy of NK cells [33, 34]. Similar polymorphism changes have also been reported in anti-myelin-associated glycoprotein (MAG) neuropathy and rheumatoid arthritis [35, 36]. Thus, the effect of the *p158* polymorphism in rituximab users is opposite to the effect in NMOSD patients who are not using this monoclonal antibody. The F/F genotype is associated with poor outcomes in rituximab users because of insufficient ADCC and depletion of B cells; in placebo mimicking natural history, the V/V genotype has the worst outcomes presumably related to increased ADCC activity perhaps with the AQP4 antibody. Either way, CD16 activity on NK/NKT cells seems to play a key in the pathogenesis of NMOSD.

**Fig. 7:**
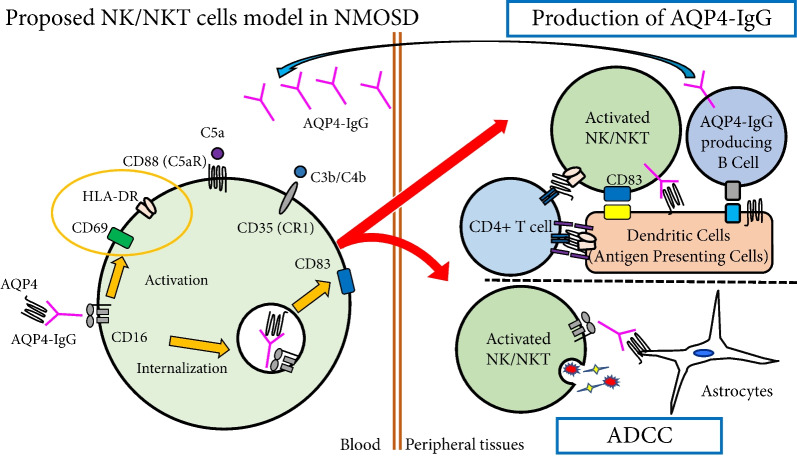
Graphical hypothesis of NK/NKT cells model in NMOSD

We speculate the following hypothesis to explain relapse initiation in NMOSD (Fig. 7): NK and NKT cells activated by systemic inflammation upregulate expression of the C5 complement receptor, CD88. These cells then take up the immune complex of the AQP4-IgG and the AQP4 molecule via CD16. The source of AQP4 may be derived from astrocytes in the central nervous system or from lungs, kidneys, and muscles, which are present in lower amounts. When inflammation destroys these tissues and AQP4 proteins are released into the blood, it binds to circulating AQP4-IgG and form immune complexes. Some reported cases that triggered NMOSD relapse, such as infection or tumor [37, 38], may support this hypothesis. NK/NKT cells that internalize these immune complexes via CD16A are activated and express CD69 and HLA-DR, then they migrate into lymph nodes where they interact with and activate AQP4-reactive T cells and B cells. Together, the adaptive immune response to AQP4 initiates an attack. Currently, the best theory for CNS localization of the AQP4 immune response is based on mouse models showing that Th17-polarized AQP4-reactive T cells adoptively transferred to wild-type mice preferentially attack the optic nerves, then the spinal cord within 12 days [39].

There are several limitations to our present study. First, the disease control group is heterogeneous. It is necessary to collect a variety of autoimmune diseases and conduct a similar study to verify whether the results are specific to NMOSD. Second, the samples used in this study are all from the remission phase and may not reflect events occurring in the acute phase. Different cell surface marker changes may be seen in NK and NKT cells in the acute phase. On the other hand, the fact that many activated NK and NKT cells express complement receptors even in the remission phase is significant enough that we can speculate that they may play a significant role in the unique pathogenesis of NMOSD. The third limitation is the difference in the sample size of each group. Since the NMOSD group has more than twice the patients included compared to the Disease and Normal Controls, the analysis might be skewed. Also, the previous analysis of *FCGR3A* polymorphisms in rituximab-treated NMOSD patients used a total of 100 patients [33]. A similar and bigger sample size could have made detecting significant differences in each parameter more sensitive.

In summary, NK and NKT cells are thought to play a major role in the pathogenesis of NMOSD by expressing complement receptors and CD16 after activation and migrating to the periphery, triggering ADCC and prolonged production of autoantibodies. Further analysis of how immune complexes, complement, and cytokines alter NK and NKT cells themselves and how they are involved in the pathogenesis of NMOSD is necessary.

## Supplementary Information

**Additional file 1: Figure S1**. The gating strategy in the NK/NKT panel of this study. After excluding doublet cells, lymphocyte subsets were extracted using FSCs and SSCs. Next, dead cells were excluded by Live/Dead staining, and nucleated cells were gated with CD45. After using CD3 and CD14, a subset of CD3-positive CD14-negative CD56-positive cells was defined as NKT cells and a subset of CD3-negative CD14-negative CD56-positive cells as NK cells for downstream analysis. For each subset, We analyzed CD16-positive, CD35-positive, CD88-positive, CD69-positive, and CD83-positive rates; for NKT cells, we also analyzed TCR Vα24-positive rates.

**Additional file 2: Figure S2**. Analysis of TCR Vα24-Jα18-positive NKT cells. The downstream analysis of NKT cells with TCR Vα24-Jα18-positive gating among the NMOSD group showed significantly higher CD83 expression against the Normal Control group (D).

**Additional file 3: Table S1**: Spearman correlation analysis for the NMOSD group between the parameters. n.s.: not significant; *: *p* < 0.05; **: *p* < 0.01; ***: *p* < 0.001; ****: *p* < 0.0001.

**Additional file 4: Table S2**: Spearman correlation analysis with and without rituximab intervention in the NMOSD group. n.s.: not significant; *: *p* < 0.01.

## Data Availability

The datasets generated and analyzed in the study are not publicly available due to local regulations concerning patient privacy. With a reasonable request, approval for data distribution will be obtained from the institutional review board of Massachusetts General Hospital, the Guthy-Jackson Charitable Foundation, and Charité-Universitätsmedizin Berlin. Then, the anonymized data will be made available by the corresponding author.

## Abbreviations

ADCC: Antibody-dependent cellular cytotoxicity
AQP4: Aquaporin-4
NMOSD: Neuromyelitis optica spectrum disorder
anti-AQP4-IgG: Anti-aquaporin-4-IgG
APC: Antigen-presenting cells
DC: Disease controls
MS: Multiple sclerosis
MOG: Myelin oligodendrocyte glycoprotein
Ig: Immunoglobulin
CNS: Central nervous system
anti-MOG-ab: Anti-myelin oligodendrocyte glycoprotein antibody
NC: Normal controls
PBMC: Peripheral blood mononuclear cells
CSF: Cerebrospinal fluid
GFAP: Glial fibrillary acidic protein
RTX: Rituximab
