# Supplementary figures and images for: Upregulated complement receptors correlate with Fc gamma receptor 3A-positive natural killer and natural killer-T cells in neuromyelitis optica spectrum disorder
Source: J Neuroinflammation. 2022 Dec 12;19:296. doi: 10.1186/s12974-022-02661-1 (PMC9743562; doi:10.1186/s12974-022-02661-1)

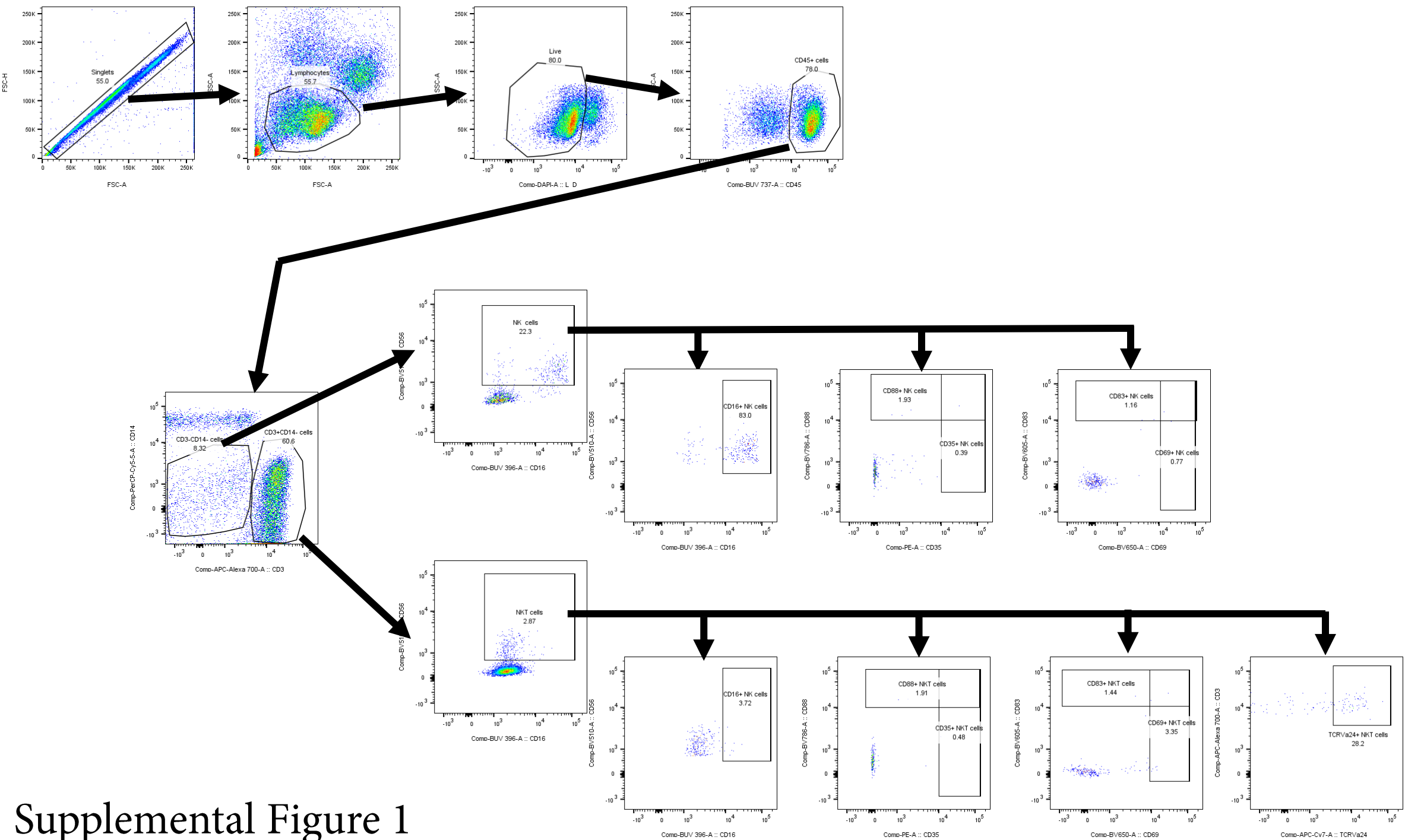

Supplemental Figure 1

Supplement: Supplementary file 1 — Additional file 1: Figure S1. The gating strategy in the NK/NKT panel of this study. After excluding doublet cells, lymphocyte subsets were extracted using FSCs and SSCs. Next, dead cells were excluded by Live/Dead staining, and nucleated cells were gated with CD45. After using CD3 and CD14, a subset of CD3-positive CD14-negative CD56-positive cells was defined as NKT cells and a subset of CD3-negative CD14-negative CD56-positive cells as NK cells for downstream analysis. For each subset, We analyzed CD16-positive, CD35-positive, CD88-positive, CD69-positive, and CD83-positive rates; for NKT cells, we also analyzed TCR Vα24-positive rates. [file 12974_2022_2661_MOESM1_ESM.pdf]

Supplemental Figure 2

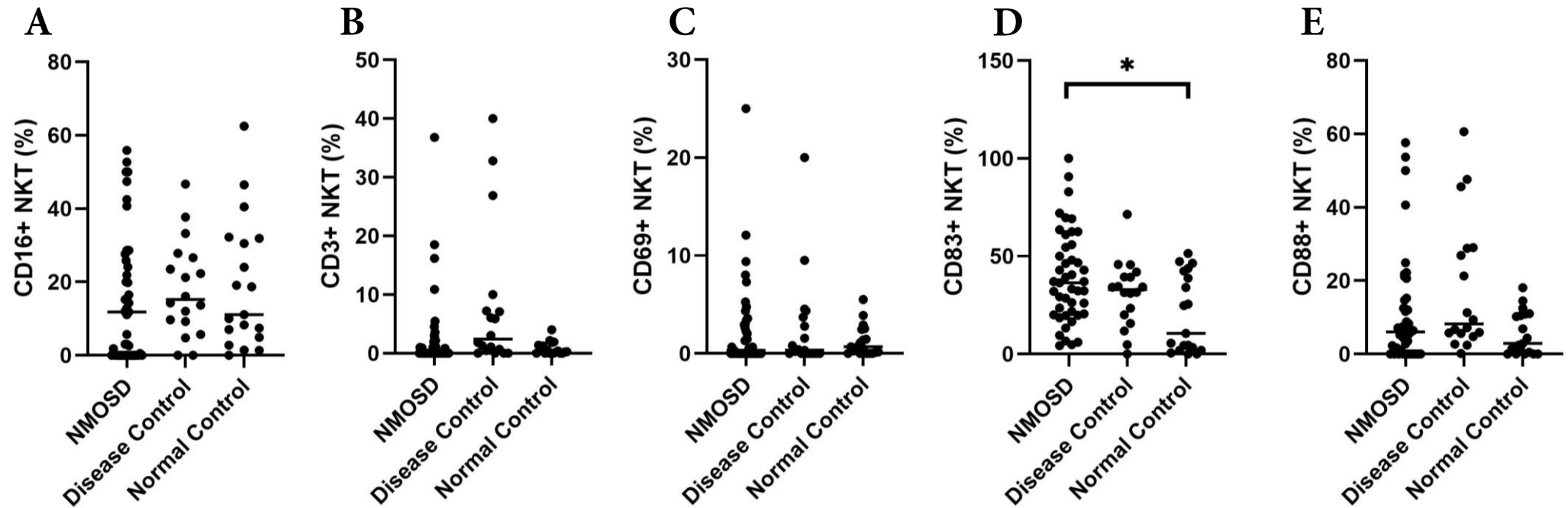

Supplement: Supplementary file 2 — Additional file 2: Figure S2. Analysis of TCR Vα24-Jα18-positive NKT cells. The downstream analysis of NKT cells with TCR Vα24-Jα18-positive gating among the NMOSD group showed significantly higher CD83 expression against the Normal Control group (D). [file 12974_2022_2661_MOESM2_ESM.pdf]
