# Supplementary material for: Upregulated complement receptors correlate with Fc gamma receptor 3A-positive natural killer and natural killer-T cells in neuromyelitis optica spectrum disorder
Source: J Neuroinflammation. 2022 Dec 12;19:296. doi: 10.1186/s12974-022-02661-1 (PMC9743562; doi:10.1186/s12974-022-02661-1)
Supplement: Supplementary file 3 — Additional file 3: Table S1: Spearman correlation analysis for the NMOSD group between the parameters. n.s.: not significant; *: p < 0.05; **: p < 0.01; ***: p < 0.001; ****: p < 0.0001. [file 12974_2022_2661_MOESM3_ESM.pdf]

(%)	NK			NKT		
	p		***r***	***p***		***r***
**CD16 vs**						
**CD35**	0.0082	**	-0.3896	0.0009	***	0.4785
**CD88**	0.0009	***	-0.4782	0.8333	ns	
**CD69**	0.0004	***	-0.5061	0.6887	ns	
**CD83**	0.1132	ns		0.0104	*	0.3784
**TCR Va24**	n/a			0.0352	*	0.3147
**CD35 vs**						
**CD88**	0.0449	*	0.3005	0.0330		0.3185
**CD69**	0.4122	ns		0.0055	**	0.4071
**CD83**	<0.0001	****	0.6482	<0.0001	****	0.7900
**TCR Va24**	n/a			<0.0001	****	0.6363
**CD88 vs**						
**CD69**	0.0004	***	0.5040	<0.0001	****	0.7103
**CD83**	0.0034	**	0.4272	0.0075	**	0.3934
**TCR Va24**	n/a			0.0252	*	0.3335
**CD69 vs**						
**CD83**	0.0648	ns		<0.0001	****	0.6218
**TCR Va24**	n/a			<0.0001	****	0.6094
**CD83 vs**						
**TCR Va24**	n/a			<0.0001	****	0.7951
