# Supplementary material for: Upregulated complement receptors correlate with Fc gamma receptor 3A-positive natural killer and natural killer-T cells in neuromyelitis optica spectrum disorder
Source: J Neuroinflammation. 2022 Dec 12;19:296. doi: 10.1186/s12974-022-02661-1 (PMC9743562; doi:10.1186/s12974-022-02661-1)
Supplement: Supplementary file 4 — Additional file 4: Table S2: Spearman correlation analysis with and without rituximab intervention in the NMOSD group. n.s.: not significant; *: p < 0.01. [file 12974_2022_2661_MOESM4_ESM.pdf]

(%)	NMOSD with RTX (n=20)	NMOSD without RTX (n=25)	*p*	
**NK Cells**	7.855±4.282	5.970±3.299	0.1532	ns
**CD16+**	50.61±18.27	38.36±19.58	0.0449	*
**CD35+**	16.31±9.469	24.54±17.10	0.0765	ns
**CD88+**	18.24±11.16	18.88±7.770	0.6550	ns
**CD69+**	12.69±7.199	13.19±8.255	0.9729	ns
**CD83+**	23.02±12.72	23.87±14.91	0.9776	ns
**NKT Cells**	5.265±2.289	6.982±5.402	0.4652	ns
**CD16+**	22.22±15.43	24.22±20.06	0.9729	ns
**CD35+**	20.27±15.99	23.53±27.28	0.4480	ns
**CD88+**	19.10±17.46	19.56±17.64	0.9234	ns
**CD69+**	17.11±13.00	18.30±13.44	0.7090	ns
**CD83+**	30.46±21.10	29.12±23.48	0.5600	ns
**TCR Vα24+**	15.59±18.65	17.22±20.68	0.7047	ns
